# Systemic immune profile in Prader-Willi syndrome: elevated matrix metalloproteinase and myeloperoxidase and reduced macrophage inhibitory factor

**DOI:** 10.1186/s13023-023-02730-5

**Published:** 2023-07-10

**Authors:** Sigrun Hope, Terje Nærland, Svein Olav Kolset, Thor Ueland, Ole A. Andreassen, Marianne Nordstrøm

**Affiliations:** 1 K.G. Jebsen Centre for Neurodevelopmental disorders, Institute of Clinical Medicine, University of Oslo, Oslo, Norway; 2 Department of Neurohabilitation, Division of Clinical Neuroscience, Oslo University Hospital, Oslo, Norway; 3 Nevsom, Department of Rare Diagnoses and Disabilities, Pediatric and Adolescent Medicine, Oslo University Hospital, Oslo, Norway; 4 Department of Nutrition, Institute of Basic Medical Sciences, University of Oslo, Oslo, Norway; 5 Research Institute of Internal Medicine, Oslo University Hospital, Oslo, Norway; 6 Institute of Clinical Medicine, University of Oslo, Rikshospitalet, Oslo, Norway; 7 K.G. Jebsen, TREC, University of Tromsø, Tromsø, Norway; 8 NORMENT: Norwegian Centre for Mental Disorders Research, Institute of Clinical Medicine, University of Oslo, Oslo, Norway; 9 Division of Mental Health and Addiction, Oslo University Hospital, Oslo, Norway; 10 Frambu Resource Centre for Rare Disorders, Siggerud, Norway; 11 Unit for Rare Neuromuscular Disorders, Movement, Muscle and Neurodegeneration, Department of Neurology, Oslo University Hospital, Oslo, Norway

**Keywords:** Prader-Willi Syndrome, 15q11-q13, Obesity, Inflammation, Cardiovascular, Extracellular matrix, Cytokines, MMP-9, Macrophage inhibitory factor, Myeloperoxidase

## Abstract

**Background:**

Prader-Willi syndrome (PWS) is a rare genetic neurodevelopmental syndrome with highly increased risk of obesity and cardiovascular disease (CVD). Recent evidence suggests that inflammation is implicated in the pathogenesis. Here we investigated CVD related immune markers to shed light on pathogenetic mechanisms.

**Methods:**

We performed a cross-sectional study with 22 participants with PWS and 22 healthy controls (HC), and compared levels of 21 inflammatory markers that reflect activity in different aspects of CVD related immune pathways and analyzed their association with clinical CVD risk factors.

**Results:**

Serum levels of matrix metalloproteinase 9 (MMP-9) was (median (range)) 121 (182) ng/ml in PWS versus 44 (51) ng/ml in HC, p = 1 × 10^-9^), myeloperoxidase (MPO) was 183 (696) ng/ml versus 65 (180) ng/ml, p = 1 × 10^-5^) and macrophage inhibitory factor (MIF) was 46 (150) ng/ml versus 121 (163) ng/ml (p = 1 × 10^-3^), after adjusting for age and sex. Also other markers tended to be elevated (OPG, sIL2RA, CHI3L1, VEGF) but not significantly after Bonferroni correction (p > 0.002). As expected PWS had higher body mass index, waist circumference, leptin, C-reactive protein, glycosylated hemoglobin (HbA1c), VAI and cholesterol, but MMP-9, MPO and MIF remained significantly different in PWS after adjustment for these clinical CVD risk factors.

**Conclusion:**

PWS had elevated levels of MMP-9 and MPO and of reduced levels of MIF, which were not secondary to comorbid CVD risk factors. This immune profile suggests enhanced monocyte/neutrophil activation, impaired macrophage inhibition with enhanced extracellular matrix remodeling. These findings warrant further studies targeting these immune pathways in PWS.

## Introduction

Prader-Willi syndrome (PWS) is a complex neurodevelopmental syndrome with a birth prevalence of approximately 1: 25.000. The syndrome is caused by lack of paternally imprinted expressed genes on chromosome 15, in the region of 15q11-q13 [1]. In addition to neurodevelopmental delay the syndrome involves hyperphagia and morbid obesity [1, 2], as well as endocrine disturbances such as growth hormone deficiency, hypogonadism, hypothyroidism and central adrenal insufficiency [3]. Over the last decades, early diagnosis, medical treatment and multidisciplinary health care offered to persons with PWS have improved [4]. Growth hormone (GH) treatment, has been available as orphan drug for PWS since 2000, and has contributed to healthier body composition. Still, cardiovascular disease (CVD) risk factors such as metabolic syndrome, type 2 diabetes and hypertension are frequent [5, 6] and their risk of pulmonary thromboembolism is twice as high as in obese persons without PWS [7]. Individuals with PWS have severely reduced life expectancy [8] and as many as 30% may not live up to adulthood [9]. Their life span is on average 32 years and CVD is among the primary causes of increased mortality [8, 9].

The main pathology underlying CVD is atherosclerosis [10] were a complex interplay between inflammation and lipid biology lead to accumulation of inflammatory cells, lipoproteins and fibrous tissue in the wall of large arteries [10, 11]. In PWS levels of the lipid hormone leptin is elevated [12], but their levels of leptin is not higher than in obese persons without PWS [13]. Studies have also reported that they have elevated systemic inflammation beyond that attributed to adiposity [14]. Still, levels of many inflammatory markers have been found to be fairly normal in PWS, as a previous study measured 41 different cytokines and only found slight increases in chemokine levels, while 37 other markers, including well known markers like TNF-alpha, IL-6 and IL-1beta were either not elevated or below the detection limit [15]. These data show the complexity of how inflammation may contribute to the high risk of CVD related mortality in PWS.

There are 18 genes within the PWS critical region, and the smallest genomic region causing Prader-Willi deletes only one gene, SNORD116 [16]. As this is a non-protein-coding RNA gene, it has been difficult to find its exact function [17]. Disruptions in other genes in the imprinted region, MAGEL2 and nectin, have been found to reduce the response to leptin [12] but how defect paternal imprinting of genes in the Prader –Willi region leads to high risk of CVD is still largely unknown. A recent genetic transcriptomic study in PWS found, however, that the main alterations involves immune activation, inflammation, extracellular matrix remodelling, and angiogenesis [18]. A systems genetics approach study found evidence of similar abnormalities, and reported that paternally imprinted genes are often co-expressed and involve genes influencing growth factor pathways, cell differentiation and extracellular matrix modeling [19].

To summarize, several lines of evidence suggest that inflammatory processes are involved in disease mechanisms underlying high risk of CVD in PWS, but it is still uncertain if there are specific inflammatory pathways that are involved in the pathogenesis. Studies suggest that that abnormally imprinted paternal genes are expressed in networks which involve extracellular matrix remodelling and growth hormone signaling, which are pathways implicated in atherosclerosis and CVD. Our objective was to investigate novel immune markers in persons with PWS versus healthy controls (HC) and explore association with comorbid CVD. We measured reliable markers reflecting different aspects of CVD related inflammation including markers of general inflammation, extracellular matrix signalling, vascular growth factors and angiogenesis, immune activation of monocyte/macrophages and neutrophils and cardiac inflammation. We aimed to adjust for established clinical CVD risk factors such as cholesterol, blood pressure, glycosylated hemoglobin (HbA1c), body mass index (BMI), Visceral Adiposity Index, waist circumference, leptin and C-reactive protein (CRP), in order to evaluate if elevations were beyond the degree of obesity. We thereby hope to provide further insight into disease mechanisms and gain new data that can potentially can be used in future studies with the aim to develop novel treatment targets for PWS.

## Methods

We performed a cross-sectional study which was approved by the Regional Committee for Medical and Health Research Ethics, Southeast Region approved the study (#2012/140).

### Participants

Patients with PWS were recruited through posting information about the study on a study-specific website and were spread in collaboration with the nationwide patient organization, using a convenient sampling. Inclusion criteria were diagnosis with PWS confirmed with genetic laboratory testing and age between 16 and 45 years. All participants with PWS had clinical diagnosis according to criteria of Holm et al. [20], and a genetic verified diagnosis. A total of 15 had a genetic deletion, 6 had maternal uniparental disomy (UPD), and one patient had unknown genetic subtype. All participants provided an informed consent to participate, together with consent from legal guardian/parent.

*Healthy controls* (HC) were randomly selected from statistical records and were invited to participate. Exclusion criteria were a history of severe medical or psychiatric diagnoses, including alcohol or illicit substance abuse, or severe mental disorders in first degree relatives.

### Clinical assessment

*Clinical diagnosis* was given according to criteria of Holm et al. [20] was obtained by trained personnel.

*Cardiovascular risk fact*ors: weight was measured twice in light clothing on a digital scale (Tanita BC-418 MA, Arlington Heights, IL, USA or Soehnle, Nassau, Germany) and recorded to the nearest 0.1 kg. Height was measured twice in an upright position with heels placed against the wall and with head fixed in Frankfurt plane using a wall-mounted stadiometer (Seca 222, Birmingham, UK) and recorded to the nearest 0.1 cm. Body mass index (BMI) was calculated using the standard formula. Waist circumference was measured twice by stretch-resistant tape (Seca, Birmingham, UK), with tape emphasized to be horizontal, and the person placed in an upright position. Measurements were taken directly on the skin, at the midpoint between the lowest rib and iliac crest after exhalation. Blood pressure was measured by trained testing personnel according to recommendations provided by the American Heart Association Council on high blood pressure research [21]. In participants with PWS an upper arm automatic blood pressure device with oscillometric measurement technique was used (Microlife BP A100 Plus, Microlife, Widnau, Switzerland). In controls resting blood pressure was measured manually by a physician under standard conditions using a sphygmomanometer (Boso, Jungingen, Germany). Mean Arterial pressure was calculated using the formula (diastolic pressure x2 + systolic pressure x1)/3. Visceral Adiposity Index (VAI) was calculated using this formula for males: VAI = WC/(39.68+(1.88*BMI)*(TG/1.03)*(1.31/HDL)) and this for females:VAI = WC / (36.58 +(1.89*BMI)*(TG/0.81)*(1.52/HDL)).

### Laboratory measures of cardiovascular risk factors

Blood samples were collected in the morning after overnight fasting and samples for plasma and serum were centrifuged within 2 h.

Fasting serum concentrations of total cholesterol, LDL cholesterol, HDL cholesterol, triglycerides and HbA1c were analyzed at the Department of Clinical Chemistry, Oslo University Hospital according to standard methods using an Integra 800 instrument (Roche Diagnostics, Basel, Switzerland). Ratios were calculated by dividing total cholesterol with HDL and triglycerides with HDL. CRP and leptin were measured together with immune marker measurements.

### Immune marker measurements

We measured a plasma levels of a broad set of immune markers, including markers of general inflammation as CRP, Pentraxin (PTX)-3 and soluble Tumor Necrosis Factor Receptor 1 (TNFR1), markers of extracellular matrix remodelling as Matrix Metalloproteinase 9 (MMP-9), Chitinase-3-Like protein 1 (CHI3L1), Tissue Inhibitor of Matrix Metalloproteinase 1 (TIMP-1), and Growth Differentiation Factor-15 (GDF15), Markers of vascular inflammation, adhesion and angiogenesis as Vascular Cell Adhesion Molecule (VCAM), Intercellular Adhesion Molecule 1 (ICAM1), E-selectin (ESEL), P-selectin (PSEL), Osteoprotegerin (OPG), Soluble Chemokine (C-X-C motif), Ligand 16 (CXCL16) and Vascular Endothelial Growth Factor (VEGF), markers of monocyte/macrophage activation as soluble Cluster of Differentiation 163 (sCD163), soluble Cluster of Differentiation 14 (sCD14) and Macrophage Inhibitory Factor (MIF), marker of T cells activation as soluble Interleukin 2 Receptor Antagonist (sIL-2RA), marker of neutrophil activation as Myeloperoxidase (MPO) and Neutrophil Gelatinase-Associated Lipocalin (NGAL) and markers of cardiac inflammation Galectin-3 (GAL3) and Interleukin 1 Receptor like 1 (IL1RL1).

These immune markers including CRP and leptin were measured in duplicate by enzyme immunoassays using commercially available antibodies (R&D Systems, Minneapolis, MN) in a 384-format using a combination of a SELMA pipetting robot (Analytik Jena AG, Jena, Germany) and a BioTek dispenser/washer (BioTek Instruments, Winooski, VT, USA). Absorption was read at 450 nm by using an enzyme immunoassay plate reader (BioTek Instruments) with wavelength correction set to 540 nm. Samples from all patients and controls were run on the same 384-well plate; the intra-assay coefficients were < 10% for all assays.

### Statistics

We evaluated if inflammatory markers were normally distributed using Kolmogorov/Smirnov test of normality and found that they were not normally distributed. To analyze differences among groups, we therefore used the Mann Whitney U test, and calculated effect sizes as Eta squared (*η*^*2*^) and Cohens d (*d)*.

Control for multiple comparisons was done by using Bonferroni corrections. As we compared 21 different mediators, the significance threshold was set to p = 0.05/21 = 0.002. Markers that was different with a p-value less than 0.002 was regarded significant.

We investigated associations between inflammatory markers and clinical cardiovascular risk factors by performing non-parametric Spearman’s Rho correlations analysis.

To adjust for possible confounders as age and gender, we used linear regression analysis. As the markers were not normally distributed and the sample size was small, we performed log transformation of immune markers, which reduces the effect of single values and secured that the residuals were normally distributed, fulfilling statistical requirements.

To control for differences in age, we also performed subgroup analysis, including only patients in age group 22–45 years, securing that mean age were similar in PWS as in HC.

We also performed subgroup analysis of PWS having normal BMI, compared with those with high BMI. We also analyzed inflammatory markers in genetic subgroups (deletion versus UPD), in **smokers**, and **in** patients who received growth hormone or sex hormones (testosterone/estrogen).

To analyze if immune markers were different in PWS independent of clinical cardiovascular factors, we performed linear regression analyses, adjusting for age, gender and all clinical CVD factors that were correlated with immune markers. We also controlled for a combined set of clinical cardiovascular markers in a regression, representing different clinical risk, obesity measures (BMI), glycosylated hemoglobin (HbA1c), and lipids (total cholesterol / HDL), and secured that collinearity was not present, with Variable Inflation Factors (VIF) < 3 for all independents. Missing values were replaced with mean.

## Results

### Demographics

As seen in Table 1, the participants with PWS (mean age 28.1 years) were younger than HC (mean age 36.5 years, p = 0.02). The frequency of females was not statistically different between groups. As expected, compared with HC, PWS had higher mean BMI, greater waist circumference, higher CRP, glycosylated hemoglobin (HbA1c), leptin, and cholesterol/HDL-ratio, while other well-known risk factors as blood pressure, total cholesterol, and triglycerides were not significantly different.

**Table 1:** Characteristics of study population

	Prader Willi syndrome n = 22	Healthy controls n = 22
Age (years) median	28.0 (6.3)**	36.5 (8.9)
Females (%)	59	32
BMI (kg/m^2^)	30.6 (6.1)**	24.0 (3.2)
Waist circumference (cm) (mean, SD)	103 (16)**	84 (10)
Leptin (mean, SD)	18 (13)**	5 (8)
C-reactive protein (mean, SD)	4.7 (4)*	0.7(2.5)
HbA1c (mean, SD) ^a^	5.7 (0.9)*	5.1 (0.2)
Total cholesterol / HDL (mean, SD)	1.2 (0.9)*	0.8 (0.4)
Total Cholesterol (mean, SD)	5.1 (0.9)	4.3 (1.2)
HDL (mean, SD)	1.2 (0.3)*	1.5 (0.4)
LDL (mean, SD)	3.4 (0.9)	3.0 (0.8)
Triglycerides / HDL^b^ (mean, SD)	4.6 (1.6)	3.3 (0.8)
Tobacco (%)^cd^	10	0
Hormone replacement (%)	55	0
Systolic BP (mean, SD)^d^	130 (18)	120 (10)
Diastolic BP (mean, SD)^d^	83 (12)	80 (9)
Mean Arterial Pressure^d^(mean, SD)	98 (13)	93 (9)
Visceral adiposity Index (mean, SD)	0.59 (0.2)	0.56 (0.2)

Data presented as mean with (standard deviation) or percent of population. *= Significantly different using p < 0.05,** = significantly different with p < 0.001); HDL = high density lipoprotein, LDL = low density lipoprotein, BP = blood pressure. Hormone replacement: growth hormone (n = 10) and testosterone/estrogen (n = 2); Cholesterol, LDL, HDL, ^a^:: missing 6, ^b^: missing 3, ^c^: one patient smoked and one used moist snuff, missing 4, ^d^: missing 4

### Levels of inflammatory markers in Prader-Willi syndrome compared with healthy controls

The levels of inflammatory markers are shown in Table 2. PWS had nominally significant higher serum levels of seven markers (p < 0.05), and after Bonferroni correction for multiple comparisons, three markers remained significant (p < 0.002). Median and range level of MMP-9 was 121 (182) ng/ml in PWS versus 44 (51) ng/ml in HC, median and range of MPO was 183 (696) ng/ml in PWS versus 65 (180) ng/ml in HC, and median and range MIF was 46 (150) ng/ml in PWS versus 121 (163) ng/ml in HC. These abnormalities had large effect sizes for MMP-9: Eta squared (η)^2^= 0.64, Cohen’s d = 2.7, p = 1 × 10^− 6^, for MPO: Eta squared η^2^ = 0.41, Cohen’s d = 1.7, p = 4 × 10^− 5^ and for MIF: Eta squared η^2^ = 0.32, Cohen’s d = 1.4, p = 2 × 10^− 4^.

**Table 2:** Median and mean serum levels of CVD markers in Prader Willi Syndrome and healthy controls

	Prader Willi Syndrome			Healthy Controls			
	Median	Mean	SD	Median	Mean	SD	Sig
MMP-9 (ng/ml)	121	127	46	44	48	16	1 × 1^− 7^
MPO (ng/ml)	183	234	167	65	71	44	4 × 10^− 5^
MIF (ng/ml)	46	68	45	121	123	46	4 × 10^− 4^
CHI3L1 (ng/ml)	31	32	12	20	23	10	0.007
sIL2RA (ng/ml)	1.08	1.15	0.48	0.75	1.12	1.13	0.02
VEGF (ng/ml)	122	283	501	94	139	163	0.03
OPG (ng/ml)	0.92	1.2	0.86	0.73	1.02	1.5	0.03
GDF15 (ng/ml)	245	273	132	203	206	51	0.05
CXCl16 (ng/ml)	4.7	4.9	2.0	3.6	3.9	1.0	0.05
sTNFR1 (ng/ml)	1.9	1.8	0.66	1.3	1.6	0.59	0.05
PTX3 (ng/ml)	0.83	0.96	0.56	0.46	1.94	4.8	0.06
sCD163 (ng/ml)	160	173	100	89	131	136	0.06
PSEL (ng/ml)	52	79	64	84	101	59	0.08
ICAM (ng/ml)	2.1	2.3	0.73	1.8	2.2	1.2	0.13
TIMP (ng/ml)	116	117	38	89	100	36	0.14
GAL3 (ng/ml)	1.11	1.35	1.24	0.90	0.96	0.42	0.23
IL1RL1 (ng/ml)	12.8	12.7	5.2	13.0	13.6	5.9	0.79
ESEL (ng/ml)	27	28	9	27	29	13	0.83
VCAM (ng/ml)	3.1	3.1	1.0	2.9	3.2	1.1	0.93
sCD14 (ug/ml)	2.4	2.6	0.64	2.3	2.4	0.58	0.19
NGAL (ng/ml)	78	99	61	69	66	28	0.10

MMP-9: Matrix Metallo Proteinase 9, MPO: Myeloperoxidase, MIF: Macrophage Inhibitory Factor, CHI3L1: Chitinase-3-Like protein 1, sIL2RA: soluble Interleukin 2 Receptor Antagonist, VEGF: Vascular Endothelial Growth Factor, OPG: Osteoprotegerin, GDF15: Growth Differentiation Factor-15CXCL16: Soluble Chemokine C-X-C motif Ligand 16, sTNFR1: soluble Tumor Necrosis Factor Receptor 1, PTX3: Pentraxin (PTX)-3, sCD163: soluble Cluster of Differentiation 163, PSEL: P-selectin, ICAM: Intercellular adhesion Molecule 1,TIMP-1: Tissue Inhibitor of Matrix Metalloproteinase 1, GAL3: Galectin-3,IL1RL1: Interleukin 1 receptor-like 1, ESEL: E-selectin, VCAM: Vascular Cell Adhesion Molecule, sCD14: soluble Cluster of Differentiation 14, NGAL: Neutrophil Gelatinase-Associated Lipocalin

**Fig. 1:**
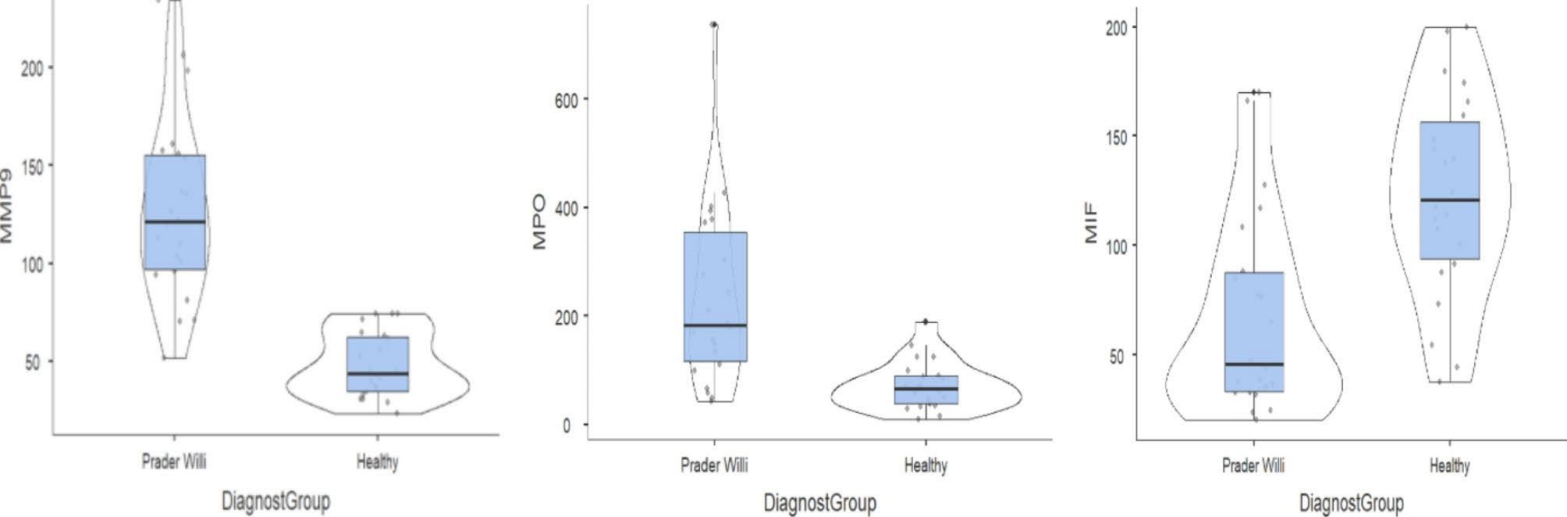
a,b,c. Violin plots showing the distribution of MMP9, MPO and MIF in Prader Willi syndrome and healthy controls MMP9: Myeloperoxidase − 9; MPO: Myeloperoxidase; MIF: Macrophage Inhibitory Factor

The distribution of MMP-9, MPO and MIF in PWS and HC is shown in Fig. 1.

### Control for confounding factors

The differences between PWS and HC were still significant after control for age and gender (p = 1 × 10^− 9^ for MMP-9, p = 0.0001 for MPO, and p = 0.001 for MIF).

#### Associations between MMP-9, MPO and MIF with cardiovascular risk factors

Correlation coefficients between MMP-9, MPO and MIF and cardiovascular risk factors are shown in Table 3. As seen, MMP-9 was significantly associated with leptin, CRP, the ratio total cholesterol/HDL, waist circumference, LDL and total cholesterol. MPO was significantly associated with levels of leptin, CRP, BMI, waist circumference and LDL, while MIF was significantly correlated with HDL. None of these markers were significantly correlated with VAI.

**Table 3:** Correlations between clinical CVD characteristics and MMP9, MPO and MIF

Cardiovascular risk factor		Inflammatory marker			
		**MMP-9**		**MPO**	**MIF**
		**r**		**r**	**r**
Total Cholesterol / HDL		0.45**	0.44**		− 0.16
HbA1c		0.49**	0.41*		− 0.11
Triglycerides		0.11	0.12		0.16
Total cholesterol		0.24	0.29		0.18
HDL		− 0.34*	− 0.22		0.33*
LDL		0.25	0.34*		0.13
Waist circumference		0.52**	0.46**		− 0.23
Body Mass Index		0.48**	0.48**		− 0.23
Triglycerides/HDL		0.23	0.22		− 0.02
Diastolic Blood Pressure		− 0.13	− 0.07		0.01
Systolic Blood pressure		0.03	0.08-		− 0.11
Mean Arterial pressure		0.07	0.03		-0.12
Leptin		0.55**	0.57**		− 0.22
Estrogen/testosterone		− 0.09	− 0.11		− 0.04
Growth hormone		− 0.02	− 0.24		− 0.07
C-reactive protein		0.45**	0.42**		− 0.26
Visceral Adiposity Index		− 0.06	− 0.10		− 0.24

*: p < 0.05; **: p < 0.01; r = Spearman’s Rho correlation coefficient; MMP-9: matrix metalloproteinase, MPO: Myeloperoxidase, MIF: Macrophage inhibitory factor. HbA1c: Glycosylated hemoglobin HDL: high density lipoprotein; LDL: low density lipoprotein; Visceral Adiposity Index: Waist circumference/(39.68+(1.88*BMI)*(TG/1.03)*(1.31/HDL)) for males and waist circumference/(36.58 +(1.89*BMI)*(TG/0.81)*(1.52/HDL)) for females

### Adjustment for clinical cardiovascular risk factors

Linear regression (Table 4) analysis showed that levels of MMP-9 and MPO were higher, and MIF was lower in PWS, also after adjusting for age, gender and a combined set of CVD related factors; HbA1c, cholesterol/HDL and BMI. In addition, we adjusted for age, gender and all clinical CVD risk factors in Table 3, and we found that levels of MMP-9, MPO and MIF were still different in PWS (p < 0.05 for all). Especially, MMP-9 was higher after adjustment for age, gender and leptin (p = 0.0007), CRP (p = 0.03), waist circumference (p < 0.001), HDL (p < 0.001), LDL (p < 0.001), total cholesterol (p = 0.03) and VAI (p < 0.001). Levels of MPO were significantly higher after adjusting for age, gender and leptin (p = 0.03), CRP (p < 0.001), LDL (p = 0.02), waist circumference (p = 0.04) and VAI (p = 0.001). Levels of MIF was lower in PWS also after adjusting for age, gender and HDL (p = 0.008), leptin (p < 0.001), CRP (p < 0.001), waist circumference (p < 0.001), and VAI (p < 0.001).

**Table 4:** Linear regression analyses showing that Prader Willi syndrome is associated with levels of MMP-9. MPO and MIF after adjustment for cardiovascular risk factors

	Log MMP-9 Unstandardized B Sig		Log MPO Unstandardized B Sig		Log MIF Unstandardized B Sig	
Constant		0.001		0.01	− 0.16	0.07
Age (years)	0.02	0.84	− 0.01	0.95	− 0.05	0.32
Gender	0.10	0.33	0.10	0.42	0.20	0.68
BMI	0.03	0.79	0.26	0.12	0.12	0.25
Lipids	0.08	0.48	0.03	0.83	0.04	0.48
HbA1c	− 0.07	0.50	− 0.04	0.73	0.80	0.78
PWS versus HC	− 0.75	0.00002	− 0.43	0.03	− 0.16	0.001

BMI: Body Mass Index; Lipids: total cholesterol / HDL; HbA1c: glycosylated hemoglobin, PWS: Prader Willi syndrome; HC: healthy controls

### Subgroup analysis

#### Comparisons of inflammation in groups with equal age

Comparisons were performed between 17 patients with PWS and 16 HC of similar mean age (32 years ± 4 versus 31 years ± 6, p = 0.41). In this subgroup we found similar results according to non-parametric tests as in the total sample: MMP-9 was higher in PWS than in HC (132 ± 51 mg/l vs. 49 ± 17 mg/l, respectively, p = 0.00001). MPO was higher in PWS than in HC (254 ± 180 mg/l vs. 72 ± 46 mg/l respectively, p = 0.001). Levels of MIF were lower in PWS than in HC (72 ± 47 mg/l vs. 125 ± 43 mg/respectively, p = 0.002). Linear regression analysis showed that these results were still significant when we adjusted for gender and BMI (p = 0.0002 for logMMP-9, p = 0.02 for logMPO and p = 0.01 for logMIF).

#### Comparisons of inflammation with respect to smoking

We performed comparisons in a subgroup were the two patients who smoked were excluded, and found that mean levels of MMP-9 was 125 mg/l, MPO was 218 mg/l and 66 mg/l in non-smoking patients, which were significantly different from healthy controls also after adjustments for age, gender, HbA1c, total cholesterol/HDL and BMI (p = 0.0005 for logMMP-9, p = 0.02 for logMPO and p = 0.009 for MIF).

#### Comparisons of inflammation in PWS with normal BMI

Patients with PWS were divided according to BMI, one group of PWS patients (n = 11) had BMI of 25.5 kg/m^2^., which was equal as in HC (24.0 kg/m^2^), and another group of PWS (n = 11) had high of BMI 37 kg/m^2^. Anova analysis showed that levels of MMP-9 and MPO were equally high in the two groups of PWS, and that both groups (both those with high BMI and normal BMI) had higher levels of MMP-9 and MPO than HC (p < 0.007 for all). MIF was equal in PWS with normal BMI versus high BMI, and PWS with normal BMI had significantly lower MIF than HC, and in PWS with high BMI there was a trend of lower MIF which was statistically non-significant (p = 0.06).

#### Comparisons within the PWS group

##### Comparisons with respect to genetic differences

There were 6 patients having uniparental disomy and 15 having a genetic deletion, and levels of MMP-9, MPO and MIF in these two groups were equal (p = 0.52, p = 0.47 and p = 0.17, respectively). Levels of all the other inflammatory markers, including leptin, were also equal (p > 0.27 for all).

##### Comparisons with respect to growth hormone replacement

There were 10 patients who received Growth hormone treatment, and 12 without treatment. Median levels of MMP-9, MPO and MIF were equal in these two groups (p = 0.92, p = 0.50, and p = 0.62 respectively).

##### Comparisons with respect to sex hormone replacement

There were only two patients receiving estrogen or testosterone, and their mean levels of MMP-9, MPO and MIF were 144 mg/l; 182 mg/l and 62 mg/l respectively, which are values within +/- 1 Standard Deviation from mean in PWS and outside the confidence intervals for mean levels in HC.

## Discussion

The main result of the current study is that patients with PWS have significant elevations of the enzymes MMP-9 and MPO and reduced levels of the cytokine MIF. Further, after controlling for cardiovascular risk factors such as BMI, glucose, and cholesterol, immune abnormalities were still significantly different, suggesting that they are characteristics of PWS and not only secondary to obesity or other cardiovascular risk factors. Levels were independent of genetic subgroup and equally high in patients treated with growth hormone, which is in line with inflammation not being significantly reduced by GH treatment [22]. Also the growth factors (CHI3L1 and VEGF), Interleukin 2Ra, and Osteoprotegerin tended to be higher in PWS (p < 0.05), although not significant after Bonferroni correction, while adhesion molecules (VCAM, ICAM, ESEL and PSEL) were in the normal range. Together, this supports the notion that pathological systemic inflammation characterizes PWS and may have a role in their increased risk of CVD.

To the best of our knowledge, this is the first time MMP-9, MPO and MIF have been investigated in PWS, and our results of higher degree of inflammation, even after control for leptin, CRP, abdominal circumference, VAI and BMI, are in accordance with studies showing that systemic inflammation is higher even in comparison with equally obese persons [14, 23].

Elevations of MMP-9 and MPO are in line with previous studies showing high levels of MMP-9 and MPO in obese individuals [24], and with that they are among the most up-regulated genes in coronary heart disease [25]. Low levels of MIF were somewhat unexpected, as MIF mainly has been described as high in CVD [26]. Still, endogenous MIF has been found to have dichotomous effects, and seems protective against aging-induced cardiac dysfunction and against negative effects of fructose on lipid metabolism [27–29] and low levels have been associated with increased risk of CVD [30].

The molecular mechanisms linking the imprinted genes in PWS‘s critical region [1] with high MMP-9 and MPO are largely unknown. However, by using a systems-level genetic approach it was shown that imprinted genes are co-expressed in a network which are enriched in extra cellular matrix and growth factor signaling [18]. And a gene expression study in PWS highlighted similar pathways including inflammation, extracellular matrix remodeling and angiogenesis [19]. In line with this, the gene SNORD116 in the PWS critical region influences the expression of the genes LIF, IFITM3 and CCL27 [31] which are implicated in inflammatory responses [32–34]. These genetic studies are in line with our findings of altered MMP-9, MPO and MIF as these markers may reflect increased activation in pathways of inflammation, extracellular matrix, growth, and angiogenesis [36, 37, 38]. We therefore hypothesize that activated MMP-9 and MPO reflect important disease mechanisms in PWS which goes beyond disrupted carbohydrate metabolism and obesity.

When it comes to cellular mechanisms, increased levels of MPO and MMP-9 are often caused by immune cell activation, especially of neutrophils and monocytes [35, 39, 40]. The accumulation of such inflammatory cells, together with lipoproteins and fibrous extracellular tissue in the vessel walls are central findings in atherosclerosis [10, 11]. Such immune cell activation has also been found in adipose tissue [40], while the adipocyte are less important for circulating levels of MMP-9 [41, 42]. MIF is unique in that it is both produced locally in tissues by immune cells counteracting cortisol’s inhibitory effect and excreted systemically as a pituitary hormone in relation to stress [43, 44] It could be speculated that the low levels are related to pituitary deficiency which characterizes PWS [45].

The study has some limitations due to a small sample size of 22 participants with PWS. Even so, about one third of the known Norwegian population of PWS in the relevant age category participated and our sample is not smaller than previous studies which included 7 and 23 participants with PWS [14, 15]. Furthermore, in contrast to a previous equally sized study [15] all our markers were reliably measured as none were below the detection limit.

A low sample size increases the risk of type II errors, and we cannot exclude that the elevations in other immune markers as CHI3L, sIL2RA, OPG and VEGF would have been statistically significant if the sample size had been larger.

Suggestions for therapeutic targets in relation to inflammatory mediators: Although MMP-9, MPO and MIF participate in inflammation, traditional anti-inflammatory medications are not efficient inhibitors. It has been tried to develop more specific MMP-9 and MPO inhibitors for treatment of inflammatory diseases and CVD, but unfortunately the usefulness of these medications in clinical practice is still unclear [46, 47]. However minocycline is a medication with well-known MMP-9 inhibitory effect [48]. Lowering MMP-9 by minocycline has been found promising in treatment of CVD [49], as in treatment of the neurodevelopmental disorder Fragile X syndrome [50, 51]. Another option for targeting the high MMP-9 levels is treatment with bumetanide. This medication is approved for heart failure and edema, and may lower MMP-9 according to animal studies [52]. Bumetanide has also been tried as treatment for tuberous sclerosis [53] which is a neurodevelopmental disorder associated with high MMP-9 [54]. High levels of MPO and MMP-9 could perhaps also be targeted by the hormone melatonin, as studies suggest a lowering effect and positive influence on CVD [55, 56]. Targeting low levels of MIF could be tried by treatment with the anti-obesity drug pioglitazone as it may reduce CVD [57, 58] and increase MIF expression [59]. Clinical trials in PWS has been used the anti-obesity medication liraglutide and the hormone oxytocin [60] as studies suggest these medications may reduce MMP-9 and improve CVD [61]. However, further studies are needed to clarify how the abnormal immune profile in PWS could be targeted.

To conclude, the present study showed elevated levels of MMP-9 and MPO and reduced levels of MIF of PWS, which are altered beyond comorbid obesity and clinical cardiovascular risk factors. The immune profile suggests involvement of specific immune abnormalities in the pathogenetic mechanisms underlying PWS, which may explain the high level of comorbid CVD. These findings warrant further studies of these immune pathways in PWS.

## Data Availability

The data that support the findings of the current study are available from the corresponding author upon reasonable request. Sharing of sensitive, individual data are restricted by GDPR regulations and Ethics requirements.
